# Interactions between magnetite and humic substances: redox reactions and dissolution processes

**DOI:** 10.1186/s12932-017-0044-1

**Published:** 2017-10-19

**Authors:** Anneli Sundman, James M. Byrne, Iris Bauer, Nicolas Menguy, Andreas Kappler

**Affiliations:** 1 Geomicrobiology, Center for Applied Geosciences, University of Tuebingen, Sigwartstrasse 10, 72076 Tuebingen, Germany; 2 Institut de Minéralogie, de Physique des Matériaux et de Cosmochimie, Sorbonne Universités, Université Pierre et Marie Curie, UMR 7590, CNRS, MNHN, IRD, 75252 Paris Cedex 05, France

**Keywords:** Magnetite, Humic substances, Redox, Dissolution, Electron transfer

## Abstract

Humic substances (HS) are redox-active compounds that are ubiquitous in the environment and can serve as electron shuttles during microbial Fe(III) reduction thus reducing a variety of Fe(III) minerals. However, not much is known about redox reactions between HS and the mixed-valent mineral magnetite (Fe_3_O_4_) that can potentially lead to changes in Fe(II)/Fe(III) stoichiometry and even dissolve the magnetite. To address this knowledge gap, we incubated non-reduced (native) and reduced HS with four types of magnetite that varied in particle size and solid-phase Fe(II)/Fe(III) stoichiometry. We followed dissolved and solid-phase Fe(II) and Fe(III) concentrations over time to quantify redox reactions between HS and magnetite. Magnetite redox reactions and dissolution processes with HS varied depending on the initial magnetite and HS properties. The interaction between biogenic magnetite and reduced HS resulted in dissolution of the solid magnetite mineral, as well as an overall reduction of the magnetite. In contrast, a slight oxidation and no dissolution was observed when native and reduced HS interacted with 500 nm magnetite. This variability in the solubility and electron accepting and donating capacity of the different types of magnetite is likely an effect of differences in their reduction potential that is correlated to the magnetite Fe(II)/Fe(III) stoichiometry, particle size, and crystallinity. Our study suggests that redox-active HS play an important role for Fe redox speciation within minerals such as magnetite and thereby influence the reactivity of these Fe minerals and their role in biogeochemical Fe cycling. Furthermore, such processes are also likely to have an effect on the fate of other elements bound to the surface of Fe minerals.

**Electronic supplementary material:**

The online version of this article (doi:10.1186/s12932-017-0044-1) contains supplementary material, which is available to authorized users.

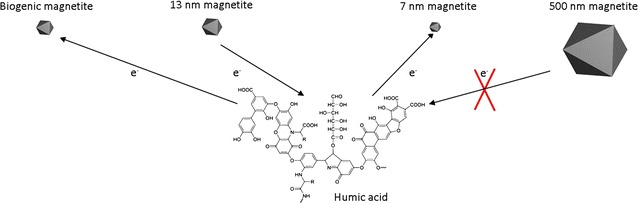

## Introduction

Iron (Fe) is a ubiquitous, redox-active element that constitutes a significant fraction of the Earth’s crust and plays an important role in controlling the fate of numerous nutrients and toxic elements [1]. Humic substances (HS) are highly abundant in aquatic and terrestrial ecosystems and can undergo a number of reactions with Fe, e.g. form complexes with both Fe(II) and Fe(III) via carboxyl groups [2] and sorb to mineral surfaces [3]. HS are also redox-active [4, 5] with multiple redox-active functional groups including quinone and phenolic groups [6–10] and can donate electrons to a number of dissolved and solid Fe(III) compounds [2, 11–15] resulting in the reduction and subsequent dissolution of minerals. Dissolved and solid-phase HS can also serve as electron acceptors or donors for microorganisms [4, 16], resulting in reduced HS whose prevalence vary with the microbial community, but is expected to be abundant in environments such as reduced sediments and water logged soils. Finally, HS can act as electron shuttles between bacteria and Fe(III) minerals in microbially mediated Fe(III) reduction [17, 18].

The capacity for HS to donate electrons to Fe(III) compounds is correlated to the reduction potential of the Fe(III) electron acceptor. Whereas HS have been shown to reduce several Fe(III) minerals, similar electron transfer reactions have not been demonstrated between humic substances and Fe(II)-containing minerals such as magnetite (Fe_3_O_4_). Magnetite has a standard redox potential of − 314 mV (for the redox couple Fe^2+^/α-Fe_3_O_4_, [Fe^2+^] = 10 µM, [19]), which is within the lower end of reported redox potentials for redox-active moieties present in HS (+ 0.15 to − 0.3 V relative to the standard hydrogen electrode [20]). The low reduction potential of magnetite suggests that magnetite can act as a good reductant but not as a good electron acceptor for electron transfer from humic substances or microorganisms although in a few cases also microbial reduction of magnetite has been described [21–23]. Additionally, it was recently shown that magnetite can be both oxidized and reduced via Fe(II)-oxidizing and Fe(III)-reducing bacteria in a cyclic manner using the magnetite as a biogeobattery [24]. Furthermore, magnetite can be oxidized during reduction of selenite [25] or chlorinated compounds [26]. Magnetite reactivity depends on Fe(II)/Fe(III) stoichiometry [27], particle size [28, 29] and the presence of organics [28, 30]. However, it is unknown whether redox reactions between magnetite and HS can occur and if HS can induce mineralogical changes in the magnetite reflected by differences in particle size, Fe(II)/Fe(III) stoichiometry or magnetic susceptibility (MS). In order to tackle these questions, we have investigated redox reactions between HS and four different types of magnetite that were synthesized in biogenic and synthetic approaches. Magnetite was incubated with native or chemically reduced HS. We followed reduction and dissolution of magnetite as well as redox changes in both aqueous Fe species and solid Fe phases over time via wet-chemical and Mössbauer spectroscopic Fe(II) and Fe(III) quantification coupled with measurements of MS. Furthermore, the solid phase magnetite was characterized using transmission electron microscopy (TEM) and micro X-ray diffraction (µXRD) to determine potential mineralogical changes during redox reactions.

## Materials and methods

### Source of HS, preparation of HS solutions and quantification of HS sorption

Pahokee Peat humic acid Reference 1R103H2 was purchased from the International Humic Substances Society (IHSS). HS stock solutions (1 g/L) were prepared freshly for each experiment following ref [31] but using 22 mM bicarbonate buffer instead of 50 mM phosphate buffer to avoid the potential formation of vivianite (Fe_3_(PO_4_)_2_·8H_2_O). The final HS concentration in the experiments was 0.6 g/L. For chemical reduction, solutions of HS were incubated with H_2_/Pd (0.5% Pd, Acros Organics) as described previously [17, 32]. HS solutions were kept in the dark throughout the experiments. Sorption of HS to magnetite was analyzed by DOC quantification (high DOC Elementar instrument, Elementar Analysensysteme GmbH, Hanau).

### Preparation and characterization of magnetite suspensions

Four different types of magnetite, of which all represent environmental magnetite, were synthesized in an anoxic glovebox. The 13 nm biogenic magnetite was synthesized according to ref [33], and the 7, 13 and 500 nm chemically synthesized magnetite particles according to refs [29], [34], and [35] respectively and characterized via µXRD and Mössbauer spectroscopy as outlined in “Magnetic and mineralogical measurements” below. Magnetite suspensions were stored in anoxic Milli-Q (MQ) H_2_O in crimped-sealed serum flasks and kept in the dark. 10 mM magnetite stocks in 22 mM bicarbonate buffer, pH 7, were prepared a minimum of 2 weeks prior to experiments since preliminary experiments (data not shown) showed significant changes in MS of the magnetite immediately after suspension in bicarbonate buffer. This effect was likely due to leaching of Fe(II) from the solid phase. The bicarbonate buffer equilibrated magnetite samples were characterized using ferrozine [36], µXRD and TEM (Table 1). The BET analysis was conducted on samples stored in anoxic Milli-Q and the surface area was analysed with a Micromeritics ASAP 2000 instrument and ASAP 2010 software. The final magnetite concentration in the experiments was ca. 4 mM Fe_3_O_4_ or about 1 g/L.

**Table 1:** Solid phase characteristics for the four types of magnetite used in the experiments

Magnetite	Fe(II)/Fe(III)^a^	Size (nm)^b^	Size (nm)^c^	BET surface area (m^2^/g)^d^
Biogenic	0.40 ± 0.01	13.6 ± 2.1	11.4	53.7
13 nm	0.37 ± 0.01	13.2 ± 2.4	12.1	64.6
7 nm	0.21 ± 0.01	7.1 ± 1.2	6.6	156.3
500 nm	0.53 ± 0.03	524 ± 156	N/A	10.7

^a^Based on ferrozine analysis of the solid phase equilibrated in bicarbonate buffer

^b^Based on TEM analysis of bicarbonate buffered sample

^c^Based on X-ray diffraction analysis of bicarbonate buffered sample

^d^On magnetite samples stored in anoxic MQ H_2_O

### Quantification of magnetite dissolution and redox changes in the presence of HS

Glassware used in the HS-magnetite experiments was acid washed and sterilized in an oven at 180 °C for 4 h. All other equipment (e.g. pipet tips and butyl stoppers) was autoclaved (121 °C). To avoid mineralogical changes, no attempts to sterilize the magnetite were employed. Magnetite dissolution and redox changes were quantified in batch experiments where anoxic magnetite suspensions were mixed with native and reduced HS solutions under anoxic conditions in a glovebox. After closing the bottles with air-tight butyl rubber stoppers and crimping, the headspace was exchanged to N_2_/CO_2_ and the bottles were placed on rolling shakers in the dark at room temperature outside of the glovebox. Control experiments were run in parallel with either HS (native and reduced) or each of the four magnetites only in order to quantify Fe(II) and Fe(III) leaching from HS or magnetite. The experiment was setup with sacrificial bottles in triplicate for each time point (0, 2, 24, 48 h, 7, 14 and 28 days). The samples were analyzed via sequential extractions at the selected time points to quantify Fe(II) and Fe(III) in the dissolved and solid phase. The liquid phase was initially separated from the solid phase, before a phosphate extraction (5 mM at pH 7.5) was conducted to remove HS from the mineral surfaces (including HS-bound Fe) to avoid HS-induced redox reactions upon acidification. Loosely bound Fe(II) was extracted by employing an acetate extraction (0.5 M, pH 4.9). All liquid samples were stabilized with 1 M anoxic HCl. The solid phase was dissolved in 6 M anoxic HCl overnight. The next day, anoxic MQ H_2_O was added to the samples before they were taken out of the glovebox since O_2_ can oxidize Fe(II) in 6 M HCl under oxic conditions [37]. All samples were analyzed for Fe(II) and Fe_tot_ by the spectrophotometric ferrozine assay [36]. The dissolved Fe concentrations reported in the manuscript hereafter are the sum of the Fe present in the supernatant, phosphate and acetate extraction. To facilitate the discrimination between dissolved and solid phase Fe, roman numerals (i.e. Fe(II) and Fe(III)) denote Fe present in the solid form while superscript (i.e. Fe^2+^ and Fe^3+^) denote Fe present in dissolved form.

### Magnetic and mineralogical measurements

The MS was measured with a KLY-3 Kappabridge device (Agico Co., Brno, Czech Republic) as described in ref [38]. The bottles were shaken vigorously before each MS measurement. The triplicate samples for MS measurements were pooled after the last measurement (i.e. after 2 months) and analyzed by µXRD and Mössbauer spectroscopy. µXRD samples were prepared by centrifuging the samples, separating the supernatant from the pellet and then drying the solid phase in an incubator (28 °C) in an anoxic glovebox. The solid samples were then ground, mounted and transported under anoxic conditions. Data was collected with a Bruker D8 Discover XRD instrument (Bruker, Germany) equipped with a Co Kα X-ray tube, (λ = 0.17,902 nm, 30 kV, 30 mA) and GADDS area detector [39]. The crystalline minerals in the samples were identified via comparison with reference samples from the International Center for Diffraction Data database. The average crystallite sizes were calculated using the Scherrer equation [40]. For each sample in the series, ^57^Fe Mössbauer spectra were obtained at 140 K with additional spectra recorded at 77 K for the 7 nm samples. Samples were prepared inside an anoxic glovebox (100% N_2_) by filtration (0.45 µm mixed cellulose esters). The filter papers loaded with sample were sealed anoxically between two layers of Kapton tape and kept in anoxic bottles until measurement. Samples were loaded into a closed cycle exchange gas cryostat. The Mössbauer spectrometer (WissEL) was operated in transmission mode, with a ^57^Co/Rh source driven in constant acceleration mode and calibrated with a 7 µm thick α-^57^Fe foil measured at room temperature, which was also used to determine the half width at half maximum (fixed to 0.128 mm/s during fitting). Fitting was carried out using Recoil (University of Ottawa) with the Voigt based fitting routine [41]. The spectra were fitted and the Fe(II)/Fe(III) ratio in the magnetite was determined based on the approach outlined by Gorski and Scherer [42].

Samples for TEM were prepared under identical conditions as the samples for ferrozine and MS analysis. High-resolution transmission electron microscope (HR-TEM) observations were performed on a JEOL 2100F microscope operating at 200 kV and equipped with a Schottky-emission gun, a high-resolution UHR pole piece, and a Gatan US4000 CCD camera. A drop containing the magnetite particles was taken from the anoxic flask using a syringe and deposited onto a carbon-coated 200 mesh copper grid. Excess water was removed with an absorbing paper and the remaining water was pumped in the airlock chamber of the microscope. Particle sizes were determined in ImageJ where the length of ca: 250 particles/sample were measured before being averaged.

## Results and discussion

### Characterization of the magnetite starting material

**Fig. 1:**
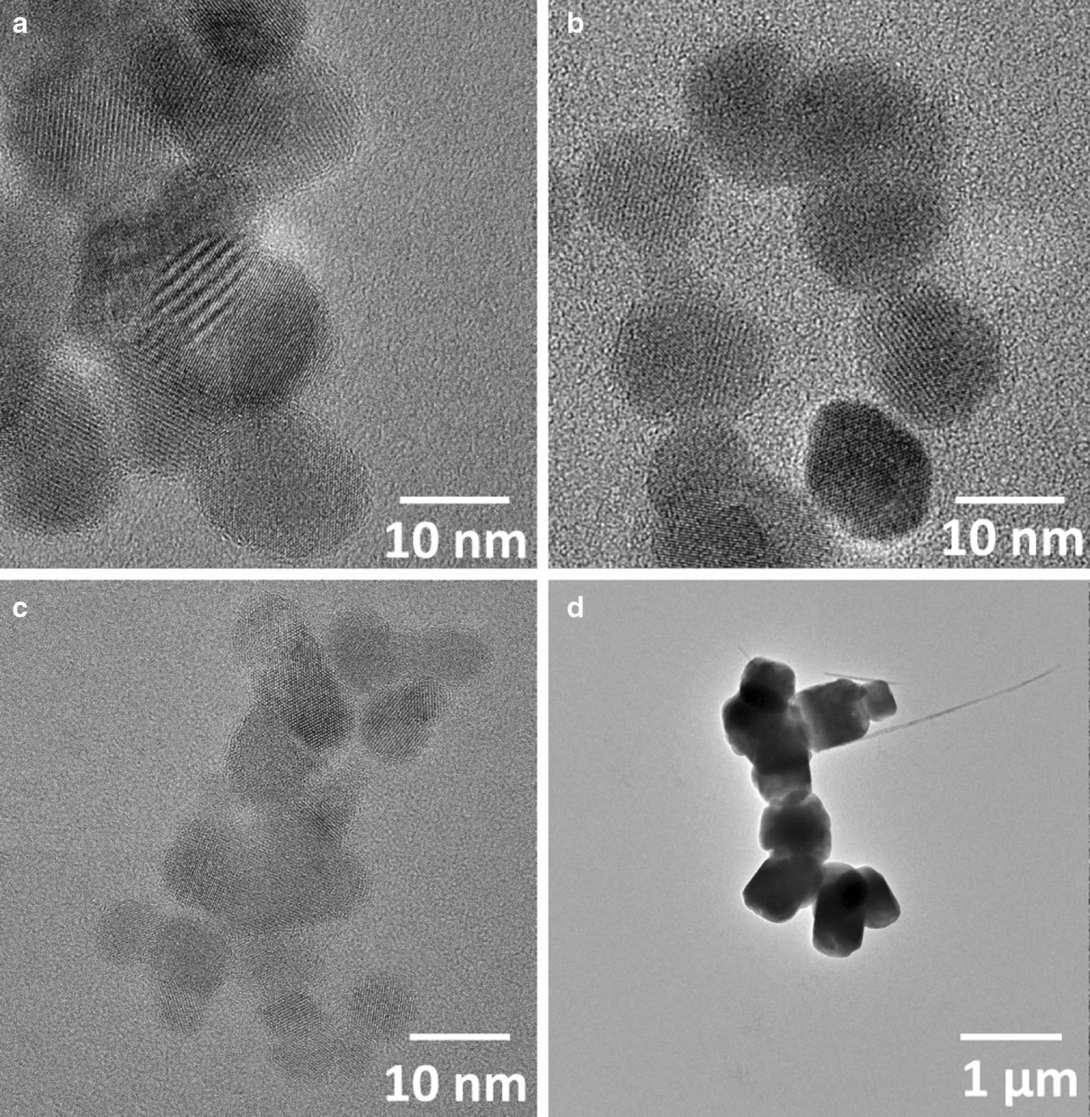
Transmission electron micrographs (bright field) of the initial magnetite particles: **a** biogenic magnetite, **b** 13 nm magnetite, **c** 7 nm magnetite, and **d** 500 nm magnetite

The magnetite starting material had particle sizes ranging from 7 to 524 nm with different Fe(II)/Fe(III) ratios (0.21–0.53) and BET surface areas between 10.7 and 156.3 m^2^/g (Table 1). The particles also varied in shape with smaller particles exhibiting spherical morphology whereas the 500 nm magnetite had a more cubic shape (Fig. 1). The biogenic magnetite, 7 nm magnetite, and 13 nm magnetite displayed similar sizes and morphologies as the particles described in the used protocols [29, 33, 34], whereas the 500 nm magnetite was larger than the particles reported by [28]. Three of the starting magnetite samples were oxidized to varying degrees relative to stoichiometric magnetite which has a Fe(II)/Fe(III) ratio of 0.5 (Table 1). Fe(II)-leaching by water as well as by rapid rinsing with an acidic solution has previously been reported [27, 28] and has been attributed to a release of surface bound Fe(II). Therefore, the pre-equilibration of the magnetite samples in anoxic bicarbonate buffer is a likely cause of the Fe(II)/Fe(III) ratios lower than 0.5. The smaller surface/volume ratio of the 500 nm magnetite probably reduced the extent of magnetite oxidation and/or Fe(II)-leaching by the bicarbonate buffer. Furthermore, the protocol for the 7 nm magnetite has been reported to produce highly oxidized magnetite particles [28]. Magnetite present in the environment may also become oxidized via exposure to bicarbonate present in the soil solutions.

### Magnetite dissolution and Fe(II)-leaching in the absence of humic substances

**Fig. 2:**
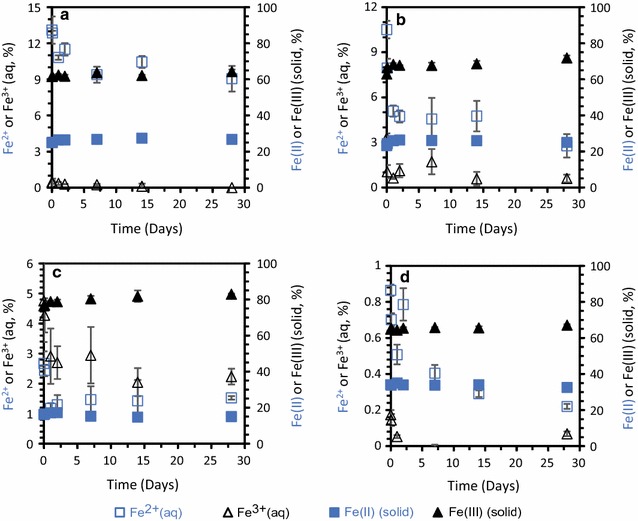
Incubation of 1 g/L of biogenic, 13, 7 and 500 nm magnetite in bicarbonate buffer. All concentrations are expressed as percentage of total Fe concentration for total aqueous Fe^2+^ (open squares), total aqueous Fe^3+^ (open triangles), solid Fe(II) (filled squares) and solid Fe(III) (filled triangles) in **a** biogenic magnetite **b** 13 nm magnetite **c** 7 nm magnetite, and **d** 500 nm magnetite. Standard deviations of all experiments were calculated from three independent parallels

Despite the pre-equilibration of magnetite in a bicarbonate buffer, further suspension of the magnetite suspensions in bicarbonate buffer resulted in an initial release of Fe(II) into solution, i.e. the formation of Fe^2+^ from the four magnetites. The initial Fe^2+^ concentrations of 84–1265 µM (0.7–17% of total Fe) dropped within the first 2 days and thereafter remained constant at 4–864 µM for the duration of the experiment (Fig. 2). The dissolved Fe^2+^ concentrations present in a 22 mM bicarbonate buffer exceeded the solubility of siderite which was observed to precipitate for the biogenic magnetite setup where the highest Fe^2+^ concentrations occurred (Additional file 1: Table S1). The Fe(II) release was most pronounced for the biogenic and 13 nm magnetite and the drop of ca. 500–800 µM Fe^2+^ and concurrent incorporation into the solid phase resulted in an apparent increase in solid-phase Fe(II)/Fe(III) ratio from 0.40 ± 0.01 (initial) to 0.43 ± 0.011 (after 2 days) and 0.37 ± 0.0062 (initial) to 0.39 ± 0.0028 (after 2 days) for the biogenic and 13 nm magnetite respectively (Additional file 1: Figure S1). The 7 nm magnetite had a drop of ca. 140 µM Fe^2+^ and a much smaller change in solid-phase Fe(II)/Fe(III) ratio (Additional file 1: Figure S1). We think that the Fe^2+^ is incorporated into the solid phase since sorbed Fe^2+^ would have been extracted with the 0.5 M NaAc used in our extraction scheme. Apart from the decrease in aqueous Fe^2+^ during the first days of experiments, the control experiments containing magnetite only (without HS), had quite stable Fe^2+^ concentrations in the range of ca. 25–250 µM except for the biogenic magnetite where the Fe^2+^ concentration was around 800 µM (Fig. 2). Poulton and Canfield [43] reported almost no dissolution of magnetite after 24 h extraction with 1 M sodium acetate at pH 4.5 whereas we observed 3–12.5% dissolution of the nanosized magnetite particles after 30 min of extraction with 0.5 M sodium acetate at pH 4.9. Furthermore, our nanoparticles could be dissolved in 1 M HCl and rapidly dissolved in 6 M HCl, whereas the 1 M hydroxylamine-HCl extraction used by Poulton and Canfield resulted in incomplete magnetite dissolution [43]. These differences might be caused by different dissolution kinetics which were much faster for the magnetite particles in this study compared with those of Poulton and Canfield. These differences highlight the size- and potential crystallinity dependent reactivity of magnetite observed in our experiments when comparing the nanoparticles with the 500 nm magnetite, which shows a similar reactivity as the natural and synthetic magnetite in the Poulton and Canfield paper [43].

### Magnetite dissolution and Fe(II)-leaching in the presence of humic substances

**Fig. 3:**
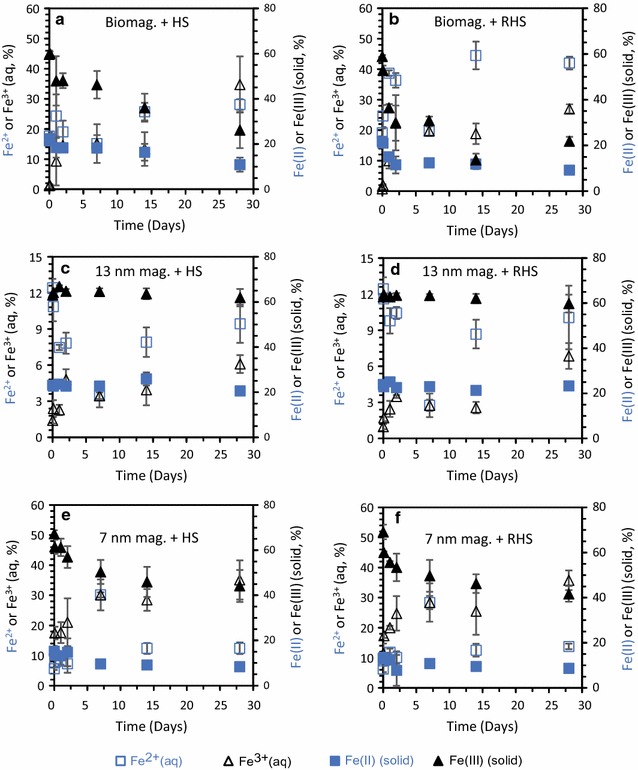
Changes in Fe concentrations during incubation of 1 g/L of biogenic, 13 nm and 7 nm magnetite with 0.6 g/L native or reduced HS. All concentrations are expressed as percentage of total Fe concentration for total aqueous Fe^2+^ (open squares), total aqueous Fe^3+^ (open triangles), solid Fe(II) (filled squares) and solid Fe(III) (filled triangles) in **a** biogenic magnetite incubated with native HS, **b** biogenic magnetite incubated with reduced HS, **c** 13 nm magnetite incubated with native HS, **d** 13 nm magnetite incubated with reduced HS, **e** 7 nm magnetite incubated with native HS, and **f** 7 nm magnetite incubated with reduced HS. Standard deviations of all experiments were calculated from three independent parallels

Control experiments with HS solutions (without magnetite) showed Fe(II)-leaching of < 40 µM (Additional file 1: Figure S2). Incubation of biogenic magnetite and 13 nm and 7 nm synthetic magnetites with native/reduced HS resulted in dissolution of the solid phase and a concurrent increase in dissolved Fe^2+^ and/or Fe^3+^ (Fig. 3). Earlier studies have shown that magnetite can be microbially reduced [23, 44], but to the best of our knowledge this is the first study showing that magnetite can also be dissolved and reduced abiotically by HS. The highest magnetite dissolution rates were observed during the first 2 days of the experiment (Fig. 3), but the dissolved Fe concentrations were still increasing by 28 days when the experiment was terminated. Most magnetite was dissolved in the setup where biogenic magnetite was incubated with reduced HS. Reduced HS has previously been reported to have a higher electron donating capacity than native HS [13]. Dissolved Fe^2+^ and Fe^3+^ increased by a total of ca. 4.8 mM over the course of the experiment and more than twice as much Fe was present in the dissolved than in the solid phase (Fig. 3b) for the biogenic magnetite reacted with reduced HS. Smaller particle sizes (i.e. 7 and 13 nm magnetite) and oxidized solid phase (i.e. 0.21 for the 7 nm magnetite, Table 1) favour mineral dissolution, but still none of the synthetic magnetite particles displayed similar magnetite dissolution as the biogenic magnetite (Fig. 3).

No dissolution was observed for the stoichiometric 500 nm magnetite (Additional file 1: Figure S4, Table S3). This is in accordance with the assumption that HS mediated magnetite dissolution is a size-dependent process with the 500 nm magnetite having the smallest specific surface area, 10.7 m^2^/g compared with 53.7–156.3 m^2^/g for the other magnetites used in these experiments (Table 1). This agrees with a recent study by Swindle et al. [28] who showed that abiotic magnetite dissolution increased with decreasing particle size in the absence of organics. However, they also suggested that organic coatings of the mineral surface protects particles from dissolution, which is in contrast to our observations. This is likely due to the large differences in magnetite concentration and the initial ratio between dissolved Fe and solid phase Fe in our study compared to that reported in Swindle et al., which is a parameter known to affect the reactivity of magnetite [27, 45, 46].

**Fig. 4:**
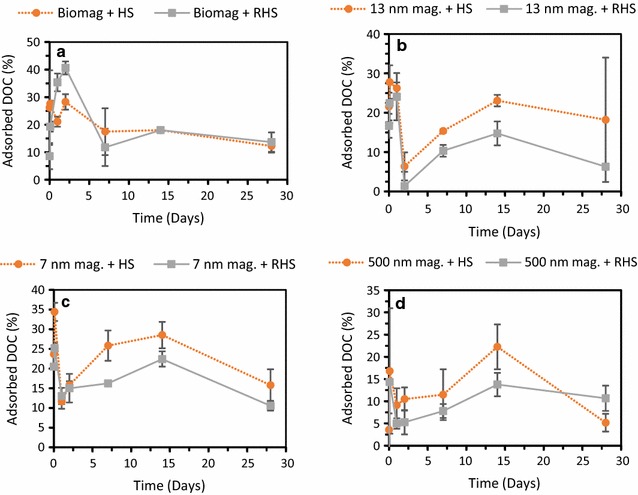
Adsorption of HS (quantified as DOC) for the four types of magnetite: **a** biogenic magnetite, **b** 13 nm magnetite, **c** 7 nm magnetite, and **d** 500 nm magnetite. The orange dashed lines and filled circles correspond to setups with native HS and the grey lines with filled squares correspond to setups with reduced HS. Standard deviations of all experiments were calculated from three independent parallels

The contribution of newly formed solid phases in our experiments during the incubation with HS was most likely minor as no other crystalline phase was detected by µXRD (Additional file 1: Figure S3). Furthermore, HR-TEM observations show that the magnetite crystallinity was conserved throughout the experiment (Additional file 1: Figure S5). However, both Fe^2+^ and Fe^3+^ form strong complexes with HS and therefore, thermodynamically driven dissolution and subsequent complexation reactions can be important pathways for the observed magnetite dissolution. The observed dissolution of magnetite particles was also supported by particle size analysis via µXRD showing a decrease in particle size over time (Additional file 1: Table S2). TEM particle size analysis also showed a weak trend with decreasing particle size over time, however, the associated standard deviations were quite big and sometimes overlapping. Interesting to note is that the level of HS adsorption does not seem to correlate with the dissolution of magnetite as there are no clear time-trends as regards the HS adsorption, which is in contrast to the time-dependent magnetite dissolution (Figs. 3, 4). Less than 50% of the HS were bound to the mineral surfaces. Therefore a plausible explanation for the observed trend, i.e. the lack of correlation between the level of HS adsorption and magnetite dissolution, is that HS molecules from solution replace HS molecules bound to mineral surfaces as both complexation in aqueous phase and sorption to mineral surfaces depend on HS properties. This exchange could lead to minor sterical hindrance and hence a higher density of sorbed HS upon dissolution and subsequent Fe(II) and Fe(III) complexation [47, 48].

### Redox reactions between magnetite and HS—characterization of the solid phase

**Fig. 5:**
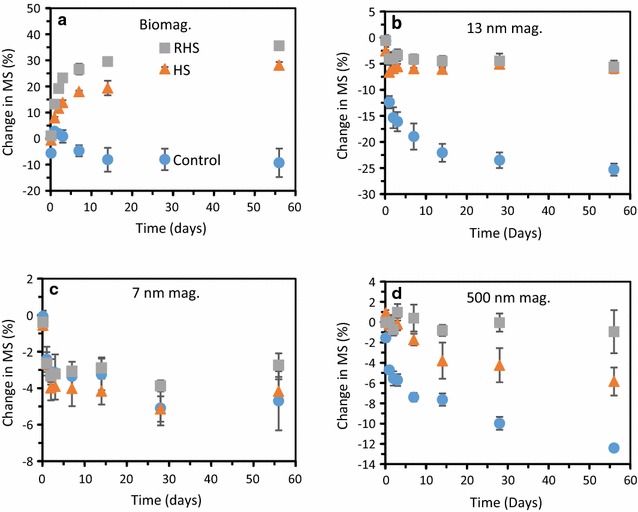
Magnetic susceptibility over time for 1 g/L of **a** biogenic magnetite, **b** 13 nm magnetite, **c** 7 nm magnetite, and **d** 500 nm magnetite in the absence of HS (blue filled circles), presence of native HS (orange filled triangles) and reduced HS (grey filled squares). Standard deviations of all experiments were calculated from three independent parallels

**Fig. 6:**
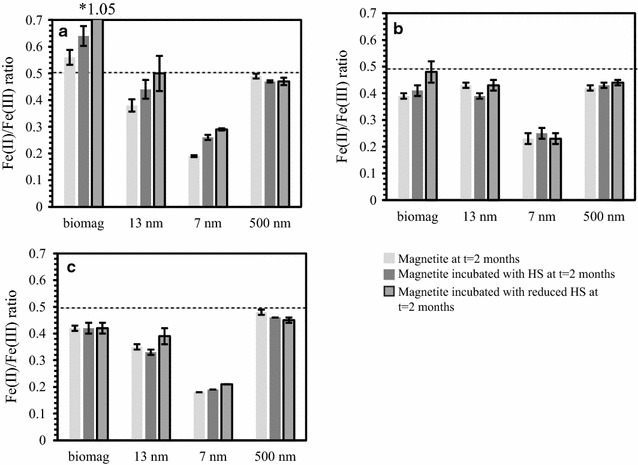
Fe(II)/Fe(III) ratios determined from ratio of chemically extracted total Fe (**a**), i.e. aqueous + solid, Fe(II)/Fe(III), Mössbauer fittings (**b**) and ferrozine analysis of solid phase Fe (**c**) of the 4 types of magnetite in the absence of HS at t = 2 months (light grey) and after 2 months incubation with native HS (dark grey) or reduced HS (dark grey with black frame)

Decreases and increases in MS have previously been linked to magnetite oxidation and reduction [24], but may also change as a result of mineral dissolution or formation of superparamagnetic particles which have higher MS than larger single domain magnetite [49]. The MS decreased in all samples except for the biogenic magnetite that was incubated with native HS and reduced HS (Fig. 5). This suggests that all other solid phases were oxidized over time, whereas the solid phase biogenic magnetite became reduced in the presence of HS and reduced HS. The solid phase Fe(II)/Fe(III) ratios determined for the 6 M HCl extracted solid phases also indicate similar oxidation and reduction of the solid phases (Table 2, Fig. 6c). The main discrepancy in the determined Fe(II)/Fe(III) ratios between the MS and ferrozine analyses is for the 13 nm magnetite incubated with reduced HS where the MS measurements indicated more or less no net redox reaction but the Fe(II)/Fe(III) ratio determined via ferrozine analysis on the 6 M HCl dissolved solid phase indicated a minor reduction of the magnetite. Furthermore, the changes in Fe concentrations and MS seemed to occur on the same time-scale in this case (Figs. 2, 3).

**Table 2:** Fe-normalized electrons transferred over 28 days relative to the redox state measured (a) directly after addition of HS or RHS, i.e. t = 0, in the HS or RHS magnetite sample and (b) t = 28 days bicarbonate control sample

Sample	meq e^−^ Fe^−1 a^	meq e^−^ Fe^−1 b^
Biogenic magnetite + HS	0.5	− 12.4
Biogenic magnetite + RHS	− 47.7	− 67.5
13 nm magnetite + HS	17.9	− 6.3
13 nm magnetite + RHS	10.7	− 16.8
7 nm magnetite + HS	0.2	− 7.8
7 nm magnetite + RHS	− 0.7	− 11.7
500 nm magnetite + HS	2.9	3.1
500 nm magnetite + RHS	2.0	2.6

Positive numbers are net-oxidation reactions and negative numbers are net-reduction reactions with respect to Fe

Using the values in Additional file 1: Table S5, the electrons transferred (z) were calculated as

$z = \frac{{\left( {\frac{{(Fe^{2 + } (\% )\,\, + \,\,Fe(II)(\% ))_{{_{ref.} }} - \,\,\,(Fe^{2 + } (\% ) + Fe(II)(\% ))_{{_{sample} }} }}{100}} \right) \times \left[ {Fe(II)} \right]\,_{tot.avg} }}{{\left[ {Fe} \right]\,\,_{tot.avg} }}$ where $\left[ {Fe(II)} \right]_{tot.\,avg} = \frac{{[Fe(II)]_{tot.ref.} + [Fe(II)]_{tot.sample} }}{2}$ and $\left[ {Fe} \right]\,_{tot.\,avg} = \frac{{\left[ {Fe_{tot} } \right]\,_{ref.} + \left[ {Fe_{tot} } \right]_{{_{sample} }} }}{2}$, Fe^2+^ represents dissolved Fe^2+^, Fe(II) represents solid phase Fe(II), [Fe(II)]_tot_ is the sum of dissolved Fe^2+^ and solid phase Fe(II), [Fe_tot_] is the total concentration of Fe (i.e. the sum of Fe^2+^, Fe(II), Fe^3+^ and Fe(III)) and ref. is (a) the t = 0 sample of the HS or RHS sample as indicated on the lines in the table or (b) the bicarbonate control at t = 28 days

The solid phase magnetite characterization using Mössbauer spectroscopy showed a satisfactory agreement with the trends already discussed, i.e. dissolution of magnetite, reduction and oxidation of solid phase and variable effects of the presence and absence of HS and/or reduced HS (Fig. 6, Additional file 1: Table S4). The Mössbauer spectra for the biogenic magnetite are characteristic of magnetite with two clear sextets corresponding to tetrahedral (A) and octahedral (B) Fe sites [50]. All starting samples exhibit similar characteristics to each other (Additional file 1: Table S1). Fitting of the data suggests that the biogenic magnetite sample incubated with reduced HS for 2 months is the most reduced sample in the series (Additional file 1: Table S4). Contrary to the µXRD which only indicated the presence of magnetite in these samples, additional doublets were present in the Mössbauer spectra for all biogenic samples corresponding to siderite, FeCO_3_. This component accounted for 1.8–5.3%. However, siderite has been reported to dissolve to a high extent in sodium acetate [43], therefore we do not expect the presence of a minor fraction of siderite to cause a large underestimation of magnetite dissolution. Among the 13 nm magnetite samples, all but the one incubated with HS show similar characteristics in their solid phase (Fig. 6, Additional file 1: Table S4). The 13 nm magnetite incubated with HS for 2 months shows an apparent decrease in the relative contribution of octahedral Fe^2.5+^ (B) site which could suggest a certain degree of oxidation which is in line with the MS results (Fig. 5 and Additional file 1: Table S4) and solid phase Fe(II)/Fe(III) analysis (Additional file 1: Figure S1). Spectra for the 7 nm magnetite collected at 140 K were not fully magnetically ordered (Additional file 1: Figure S8) and indicated that the particles were superparamagnetic due to their small particle size. However, spectra recorded at 77 K were also not fully magnetically ordered. The ca. 10% increase of the poorly defined third sextet at 140 K upon aging in the presence and absence of native and reduced HS suggests that the particles dissolved which lead to a smaller particle size for the 7 nm magnetite. This finding is in line with the other analyses (Fig. 3). Finally, all spectra for the 500 nm magnetite appear to be very similar, except for the 2 months native magnetite sample which appears to be slightly more oxidized than the others and this is also supported by our other analyses. Despite the fact that µXRD suggests the presence of goethite, no clear sextet corresponding to this mineral could be observed (Additional file 1: Figure S6). The amount of goethite in the sample must be very minor given the limited reactivity in these set-ups compared with previous studies [13].

### Redox reactions between magnetite and HS—overall redox changes

The overall redox changes cannot be concluded by only considering changes in the magnetite solid phases as they do not consider dissolution of magnetite and formation of dissolved Fe-HS complexes. Therefore, in order to elucidate the overall redox changes in the systems Fe(II) and Fe(III) concentrations in both dissolved and solid phase needs to be considered (Fig. 6a, Additional file 1: Table S3). The total (solid + dissolved) Fe(II)/Fe(III) ratios were higher than the solid Fe(II)/Fe(III) ratios as a consequence of high dissolved Fe^2+^ and Fe^3+^ concentrations (Fig. 6). The overall increase in the summed dissolved and solid phase Fe(II)/Fe(III) observed for biogenic, 13 and 7 nm magnetite reacted with native HS and reduced HS compared with bicarbonate buffer control samples indicates that the overall reaction is a reduction of Fe(III) (Fig. 6a, Table 2). However, the solid phases did not undergo such extensive reduction and the 13 nm magnetite incubated with native HS became more oxidized compared with the bicarbonate control (Fig. 6b, c). Hence, under some conditions there is a discrepancy between the overall redox reaction and the reactions of the solid phase (Fig. 6). As expected, experiments with reduced HS typically resulted in a higher net-reduction of the magnetite relative to their bicarbonate control sample compared with their native HS counterpart (Table 2). Furthermore, as previously observed for magnetite dissolution, the magnitude of redox reactions between HS and biogenic and 13 nm magnetite was different despite similar initial Fe(II)/Fe(III) stoichiometry, slightly larger particle size and larger BET surface area. Finally, the 500 nm magnetite incubation with native HS and reduced HS resulted in a minor overall oxidation and inconclusive changes in the solid phase (Fig. 6). As suggested before, there is a clear link between surface area (i.e. particle size) and reactivity in terms of electron transfer and dissolution (Table 2, Additional file 1: Figures S1 and S4).

Previous studies have shown that the amount of electrons transferred from reduced HS to Fe(III) minerals decreases with decreasing *E*_h_ values of the Fe(III) compounds (i.e., in the order 2-line ferrihydrite > goethite > hematite) [9]. Furthermore, only Fe(III) citrate and 2-line ferrihydrite have been shown to be reduced by non-reduced HS. Approximately 68 meq e^−^ Fe^−1^ were accepted when biogenic magnetite was incubated with reduced HS (Table 2). All magnetite samples, except those with 500 nm magnetite, accepted electrons from native and reduced HS when compared with the 28 day bicarbonate control samples (Table 2). In contrast, most samples showed a net donation of electrons from magnetite to HS when compared with their respective t = 0 starting samples. This discrepancy is a further support for our conclusion that the bicarbonate buffer oxidizes the magnetite over time by leaching Fe^2+^ from the solid phase. The mM range production of dissolved Fe^2+^ from magnetite (Fig. 3) might be due to an underestimation of the *E*_*h*_ value of magnetite, i.e. as discussed in Gorski [51] and/or an effect of coupled equilibrium reaction, e.g. formation of new solid phases (e.g. siderite) and complexes (Fe^2+^- and/or Fe^3+^-HS complexes). Another reason for the HS-mediated magnetite dissolution despite the low *E*_h_ of magnetite compared with e.g. ferrihydrite could be a heterogeneous distribution of Fe(II) within the magnetite, i.e. the surface is more oxidized than the bulk fraction of the magnetite with the oxidized layer reaching a depth of several nm as it was shown by Nedkov et al. [52]. Mössbauer analysis of the magnetite carried out in our laboratory showed the presence of magnetite, but the presence of a maghemite surface layer could not be verified with this technique or with µXRD. A more surface-sensitive method such as integrated low-energy electron Mössbauer spectroscopy [52] or X-ray Magnetic Circular Dichroism at Fe *L*_2,3_ edges [53, 54] would provide more information. Another likely explanation for the high magnetite dissolution is surface loading of Fe(II) from dissolved Fe(II). This hypothesis is supported by the relatively more reduced solid phases and the overall net Fe reduction observed for the biogenic and 7 nm magnetite, which were the two samples that dissolved the most. Our results suggest that mere predictions of the outcome of redox reactions between magnetite and HS based on bulk thermodynamic data have to be made with caution and that other factors such as surface processes, where the reactions actually take place, have to be taken into account. Redox-active metal impurities present in HS could have been involved in electron transfer processes between HS and magnetite. However, due to the harsh purification procedures of HS and the resulting low metal concentrations from the IHSS (including HF treatment) we believe that these processes did not influence our results significantly. This is discussed in more detail in Bauer and Kappler [13].

## Conclusions

Our study suggests that magnetite reduction and dissolution by native and reduced humic substances has to be considered as an important electron transfer pathway in anoxic environments such as sediments or waterlogged soils and has the potential to contribute to the environmental iron cycle. These reactions are likely influenced by microorganisms since they can utilize HS as electron donors and acceptors. These abiotic reactions may play an important role in environments or sites where the microbial access to mineral surfaces are physically hindered. Furthermore, the current study highlights the variability in magnetite reactivity based on the route of synthesis, i.e. abiotic or biogenic, and the resulting magnetite properties (Fe(II)/Fe(III) stoichiometry and particle size). More specifically, the high reactivity of biogenic magnetite and its propensity to be reduced and dissolved by HS indicates that magnetite of biogenic origin potentially plays a larger role in the mobilization of sorbed nutrients and toxic elements in organic rich environments compared with abiotically formed magnetite. We believe that the high reactivity of biogenic magnetite is linked to its high organic carbon content (EPS and other cell-derived biomolecules) as organic molecules have previously been linked to electron shuttling and reductive dissolution of Fe-minerals [11–15]. Furthermore, the higher solubility, i.e. reactivity, of biogenic magnetite results in dissolved Fe^2+^ which can reload the solid phase magnetite and thereby increase its propensity to dissolve. These results also have clear implications for the use of magnetite for remediation purposes: HS-induced dissolution of magnetite may result in remobilization of previously sorbed contaminants and the observed high reactivity of biogenic magnetite may indicate that it is even more suitable for redox-based remediation of contaminants such as Cr(VI).

## Additional file

**Additional file 1: Figure S1.** Results from solid phase Fe(II)/Fe(III) ratios. **Figure S2**. Leaching of Fe from HS. **Figure S3**. X-ray diffractograms of magnetite samples. **Figure S4**. Results of 500 nm magnetite incubation with HS and reduced HS. **Figure S5**. HR-TEM micrographs of selected magnetite samples. **Figure S6**. Results from Mössbauer spectroscopy fittings. **Table S1**. Results from Mössbauer fittings. **Table S2**. Results from particle size analysis by μXRD and TEM. **Table S3**. Compilation of dissolved and solid phase Fe(II) and Fe(III) for initial and end samples. **Table S4**. Results from Mössbauer fittings. **Table S5**. Fe^2+^ and Fe(II) concentrations for electron transfer calculations.

## Abbreviations

Fe: iron
HR-TEM: high-resolution transmission electron microscope
HS: humic substances
IHSS: International Humic Substances Society
MQ: Milli-Q
MS: magnetic susceptibility
SI: supporting information
TEM: transmission electron microscopy
µXRD: micro X-ray diffraction

## References

[1] Borch T, Kretzschmar R, Kappler A, Cappellen PV, Ginder-Vogel M, Voegelin A (2010). Biogeochemical Redox Processes and their Impact on contaminant dynamics. Environ Sci Technol.

[2] Fimmen RL, Cory RM, Chin Y-P, Trouts TD, McKnight DM (2007). Probing the oxidation–reduction properties of terrestrially and microbially derived dissolved organic matter. Geochim Cosmochim Acta.

[3] Tipping E (1981). The adsorption of aquatic humic substances by iron oxides. Geochim Cosmochim Acta.

[4] Lovley DR, Coates JD, Blunt-Harris EL, Phillips EJP, Woodward JC (1996). Humic substances as electron acceptors for microbial respiration. Nature.

[5] Lovley DR, Fraga JL, Coates JD, Blunt-Harris EL (1999). Humics as an electron donor for anaerobic respiration. Environ Microbiol.

[6] Ratasuk N, Nanny MA (2007). Characterization and Quantification of Reversible Redox Sites in Humic Substances. Environ Sci Technol.

[7] Aeschbacher M, Sander M, Schwarzenbach RP (2010). Novel electrochemical approach to assess the redox properties of humic substances. Environ Sci Technol.

[8] Aeschbacher M, Graf C, Schwarzenbach RP, Sander M (2012). Antioxidant properties of humic substances. Environ Sci Technol.

[9] Dunnivant FM, Schwarzenbach RP, Macalady DL (1992). Reduction of substituted nitrobenzenes in aqueous solutions containing natural organic matter. Environ Sci Technol.

[10] Curtis GP, Reinhard M (1994). Reductive Dehalogenation of hexachloroethane, carbon tetrachloride, and bromoform by anthrahydroquinone disulfonate and humic acid. Environ Sci Technol.

[11] Scott DT, McKnight DM, Blunt-Harris EL, Kolesar SE, Lovley DR (1998). Quinone moieties act as electron acceptors in the reduction of humic substances by humics-reducing microorganisms. Environ Sci Technol.

[12] Bauer M, Heitmann T, Macalady DL, Blodau C (2007). Electron transfer capacities and reaction kinetics of peat dissolved organic matter. Environ Sci Technol.

[13] Bauer I, Kappler A (2009). Rates and extent of reduction of Fe(III) compounds and O_2_ by humic substances. Environ Sci Technol.

[14] Struyk Z, Sposito G (2001). Redox properties of standard humic acids. Geoderma.

[15] Kappler A, Benz M, Schink B, Brune A (2004). Electron shuttling via humic acids in microbial iron(III) reduction in a freshwater sediment. FEMS Microbiol Ecol.

[16] Coates JD, Ellis DJ, Blunt-Harris EL, Gaw CV, Roden EE, Lovley DR (1998). Recovery of humic-reducing bacteria from a diversity of environments. Appl Environ Microbiol.

[17] Kappler A, Benz M, Schink B, Brune A (2004). Electron shuttling via humic acids in microbial iron(III) reduction in a freshwater sediment. FEMS Microbiol Ecol.

[18] Roden EE, Kappler A, Bauer I, Jiang J, Paul A, Stoesser R (2010). Extracellular electron transfer through microbial reduction of solid-phase humic substances. Nat Geosci..

[19] Thamdrup B, Schink B (2000). Bacterial manganese and iron reduction in aquatic sediments. Adv Microb Ecol 16.

[20] Aeschbacher M, Vergari D, Schwarzenbach RP, Sander M (2011). Electrochemical analysis of proton and electron transfer equilibria of the reducible moieties in humic acids. Environ Sci Technol.

[21] Dong H, Fredrickson JK, Kennedy DW, Zachara JM, Kukkadapu RK, Onstott TC (2000). Mineral transformations associated with the microbial reduction of magnetite. Chem Geol.

[22] Schütz MK, Bildstein O, Schlegel ML, Libert M (2015). Biotic Fe(III) reduction of magnetite coupled to H_2_ oxidation: implication for radioactive waste geological disposal. Chem Geol.

[23] Kostka JE, Nealson KH (1995). Dissolution and reduction of magnetite by bacteria. Environ Sci Technol.

[24] Byrne JM, Klueglein N, Pearce C, Rosso KM, Appel E, Kappler A (2015). Redox cycling of Fe(II) and Fe(III) in magnetite by Fe-metabolizing bacteria. Science.

[25] Scheinost AC, Charlet L (2008). Selenite Reduction by mackinawite, magnetite and siderite: XAS characterization of nanosized redox products. Environ Sci Technol.

[26] Heijman CG, Holliger C, Glaus MA, Schwarzenbach RP, Zeyer J (1993). Abiotic reduction of 4-chloronitrobenzene to 4-chloroaniline in a dissimilatory iron-reducing enrichment culture. Appl Environ Microbiol.

[27] Gorski CA, Scherer MM (2009). Influence of magnetite stoichiometry on feii uptake and nitrobenzene reduction. Environ Sci Technol.

[28] Swindle AL, Madden ASE, Cozzarelli IM, Benamara M (2014). Size-dependent reactivity of magnetite nanoparticles: a field-laboratory comparison. Environ Sci Technol.

[29] Vikesland PJ, Heathcock AM, Rebodos RL, Makus KE (2007). Particle size and aggregation effects on magnetite reactivity toward carbon tetrachloride. Environ Sci Technol.

[30] Swindle AL, Cozzarelli IM, Elwood Madden AS (2015). Using chromate to investigate the impact of natural organics on the surface reactivity of nanoparticulate magnetite. Environ Sci Technol.

[31] Jiang J, Kappler A (2008). Kinetics of microbial and chemical reduction of humic substances: implications for electron shuttling. Environ Sci Technol.

[32] Benz M, Schink B, Brune A (1998). Humic acid reduction by *Propionibacterium freudenreichii* and other fermenting bacteria. Appl Environ Microbiol.

[33] Byrne JM, Telling ND, Coker VS, Pattrick RAD, Gvd Laan, Arenholz E (2011). Control of nanoparticle size, reactivity and magnetic properties during the bioproduction of magnetite by Geobacter sulfurreducens. Nanotechnology..

[34] Pearce CI, Qafoku O, Liu J, Arenholz E, Heald SM, Kukkadapu RK (2012). Synthesis and properties of titanomagnetite (Fe3 − xTixO4) nanoparticles: a tunable solid-state Fe(II/III) redox system. J Colloid Interface Sci.

[35] Schwertmann U, Cornell RM (2000). Iron Oxides in the Laboratory: Preparation and Characterization.

[36] Stookey LL (1970). Ferrozine—a new spectrophotometric reagent for iron. Anal Chem.

[37] Porsch K, Kappler A (2011). FeII oxidation by molecular O_2_ during HCl extraction. Environ Chem.

[38] Porsch K, Dippon U, Rijal ML, Appel E, Kappler A (2010). In-situ magnetic susceptibility measurements as a tool to follow geomicrobiological transformation of fe minerals. Environ Sci Technol.

[39] Berthold C, Bjeoumikhov A, Brugamann L (2009). Fast XRD2 microdiffraction with focusing X-ray microlenses. Part Part Syst Charact.

[40] Patterson AL (1939). The scherrer formula for X-ray particle size determination. Phys Rev.

[41] Rancourt DG, Ping JY (1991). Voigt-based methods for arbitrary-shape static hyperfine parameter distributions in Mössbauer spectroscopy. Nucl Instrum Methods Phys Res Sect B.

[42] Gorski CA, Scherer MM (2010). Determination of nanoparticulate magnetite stoichiometry by Mössbauer spectroscopy, acidic dissolution, and powder X-ray diffraction: a critical review. Am Miner.

[43] Poulton SW, Canfield DE (2005). Development of a sequential extraction procedure for iron: implications for iron partitioning in continentally derived particulates. Chem Geol.

[44] Dong HL, Fredrickson JK, Kennedy DW, Zachara JM, Kukkadapu RK, Onstott TC (2000). Mineral transformation associated with the microbial reduction of magnetite. Chem Geol.

[45] Gorski CA, Nurmi JT, Tratnyek PG, Hofstetter TB, Scherer MM (2010). Redox behavior of magnetite: implications for contaminant reduction. Environ Sci Technol.

[46] Latta DE, Gorski CA, Boyanov MI, O’Loughlin EJ, Kemner KM, Scherer MM (2012). Influence of magnetite stoichiometry on UVI reduction. Environ Sci Technol.

[47] Fujii M, Imaoka A, Yoshimura C, Waite TD (2014). Effects of molecular composition of natural organic matter on ferric iron complexation at circumneutral pH. Environ Sci Technol.

[48] Chassé AW, Ohno T, Higgins SR, Amirbahman A, Yildirim N, Parr TB (2015). Chemical force spectroscopy evidence supporting the layer-by-layer model of organic matter binding to iron (oxy)hydroxide mineral surfaces. Environ Sci Technol.

[49] Mullins CE (1977). Magnetic susceptibility of the soil and its significance in soil science—a review. J Soil Sci.

[50] Murad E (2010). Mössbauer spectroscopy of clays, soils and their mineral constituents. Clay Min..

[51] Gorski CA. Redox behavior of magnetite in the environment: moving towards a semiconductor model. PhD thesis, University of Iowa; 2009.

[52] Nedkov I, Merodiiska T, Slavov L, Vandenberghe RE, Kusano Y, Takada J (2006). Surface oxidation, size and shape of nano-sized magnetite obtained by co-precipitation. J Magn Magn Mater.

[53] Brice-Profeta S, Arrio MA, Tronc E, Menguy N, Letard I, dit Moulin C (2005). Magnetic order in—nanoparticles: a XMCD study. J Magn Magn Mater.

[54] Carvallo C, Sainctavit P, Arrio M-A, Menguy N, Wang Y, Ona-Nguema G (2008). Biogenic vs. abiogenic magnetite nanoparticles: a XMCD study. Am Miner.

