# Supplementary material for: Interactions between magnetite and humic substances: redox reactions and dissolution processes
Source: Geochem Trans. 2017 Oct 19;18:6. doi: 10.1186/s12932-017-0044-1 (PMC5648731; doi:10.1186/s12932-017-0044-1)
Supplement: Supplementary file 1 — Additional file 1: Figure S1. Results from solid phase Fe(II)/Fe(III) ratios. Figure S2. Leaching of Fe from HS. Figure S3. X-ray diffractograms of magnetite samples. Figure S4. Results of 500 nm magnetite incubation with HS and reduced HS. Figure S5. HR-TEM micrographs of selected magnetite samples. Figure S6. Results from Mössbauer spectroscopy fittings. Table S1. Results from Mössbauer fittings. Table S2. Results from particle size analysis by μXRD and TEM. Table S3. Compilation of dissolved and solid phase Fe(II) and Fe(III) for initial and end samples. Table S4. Results from Mössbauer fittings. Table S5. Fe2+ and Fe(II) concentrations for electron transfer calculations. [file 12932_2017_44_MOESM1_ESM.docx]

Additional Information

Interactions between magnetite and humic substances: redox reactions and dissolution processes

Anneli Sundman^†^*, James M. Byrne^†^, Iris Bauer^†^, Nicolas Menguy^‡^ and Andreas Kappler^†^

^†^ *Geomicrobiology, Center for Applied Geosciences, University of Tuebingen, Sigwartstrasse 10, 72076, Germany*

^‡^ *Institut de Minéralogie, de Physique des Matériaux et de Cosmochimie, Sorbonne Universités, Université Pierre et Marie Curie, UMR 7590 CNRS, MNHN, IRD, 75252 Paris Cedex 05, France*

* corresponding author

[sundman.anneli@gmail.com](mailto:anneli.sundman@ifg.uni-tuebingen.de), [james.byrne@uni-tuebingen.de](mailto:james.byrne@uni-tuebingen.de), [iris44@web.de](mailto:iris44@web.de), [Nicolas.Menguy@impmc.jussieu.fr](javascript:void(window.open('/imp/dynamic.php?page=compose&to=Nicolas.Menguy%40impmc.jussieu.fr&popup=1','','width=820,height=610,status=1,scrollbars=yes,resizable=yes'))), [andreas.kappler@uni-tuebingen.de](mailto:andreas.kappler@uni-tuebingen.de)

This supporting information is 12 pages long and contains 6 Figures and 5 Tables.


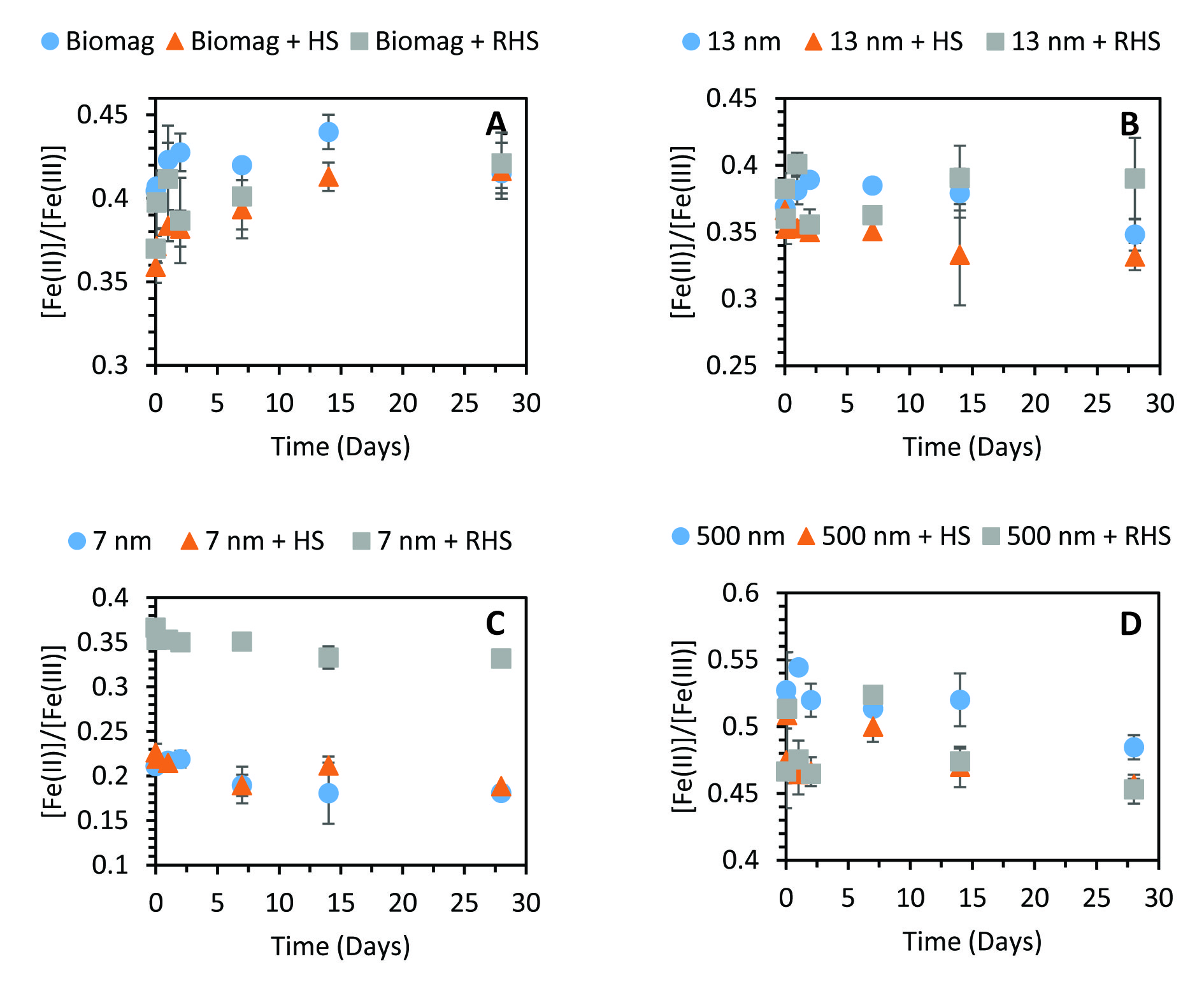


**Figure S1.** Solid phase Fe(II)/Fe(III) in the absence of HS (filled circles), presence of native HS (filled triangles) and presence of reduced HS (filled squares) for A) biogenic magnetite, B) 13 nm magnetite, C) 7 nm magnetite and D) 500 nm magnetite. Standard deviations of all experiments were calculated from three independent parallels.


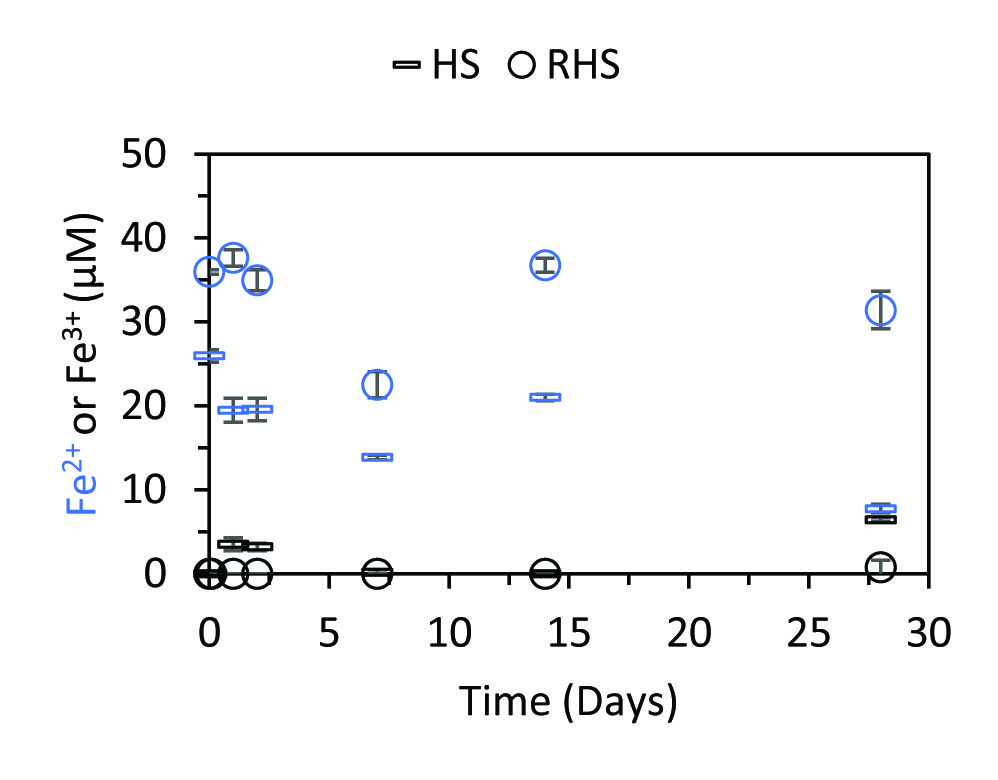


**Figure S2.** Release of Fe^2+^ and Fe^3+^ from native (HS) and chemically reduced HS (RHS) solutions (in bicarbonate buffer) into aqueous phase over time. The blue symbols refer to Fe(II) concentrations and the black symbols to Fe(III) concentrations. Standard deviations of all experiments were calculated from three independent parallels.


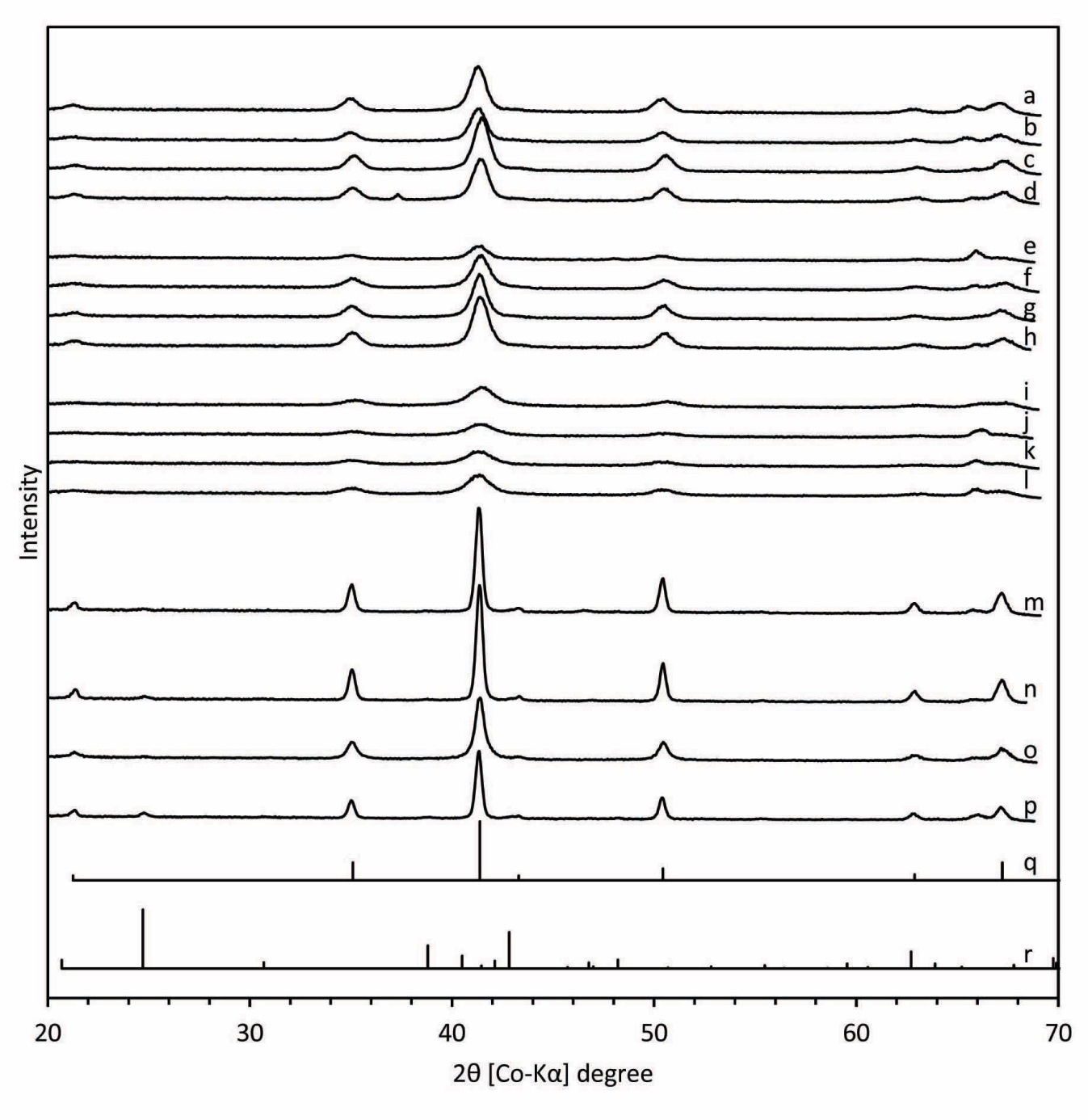


**Figure S3.** Non-processed (i.e. not background subtracted) X-ray diffractograms of a) biomag t=0, b) biomag t=2 months, c) biomag HS t=2 months, d) biomag RHS t=2 months, e) 13 nm magnetite t=0, f) 13 nm magnetite t=2 months, g) 13 nm magnetite HS t=2 months, h) 13 nm magnetite RHS t=2 months, i) 7 nm magnetite t=0, j) 7 nm magnetite t=2 months, k) 7 nm magnetite HS t=2 months, l) 7 nm magnetite RHS t=2 months, m) 500 nm magnetite t=0, n) 500 nm magnetite t=2 months, o) 500 nm magnetite HS t=2 months, p) 500 nm magnetite RHS t=2 months, q) magnetite reference and r) goethite reference.
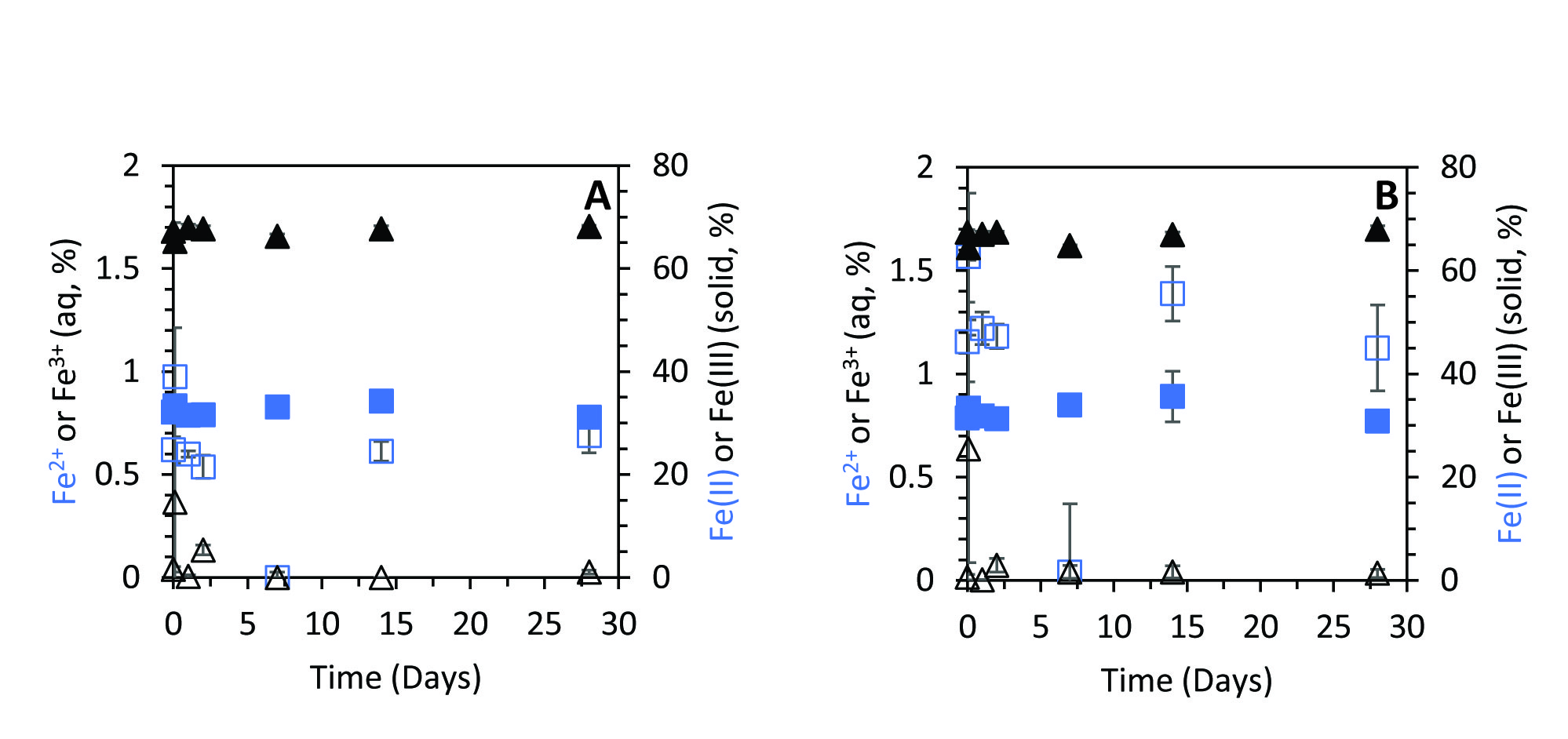


**Figure S4.** Concentration of total aqueous Fe^2+^ (open squares), total aqueous Fe^3+^ (open triangles), solid Fe(II) (filled squares) and solid Fe(III) (filled triangles) in A) 500 nm magnetite incubated with native HS and B) 500 nm magnetite incubated with reduced HS. For all experiments, 500 nm magnetite (1 g/L) was incubated with 0.6 g/L native or reduced HS. Standard deviations of all experiments were calculated from three independent parallels.

biogenic

13 nm

7 nm


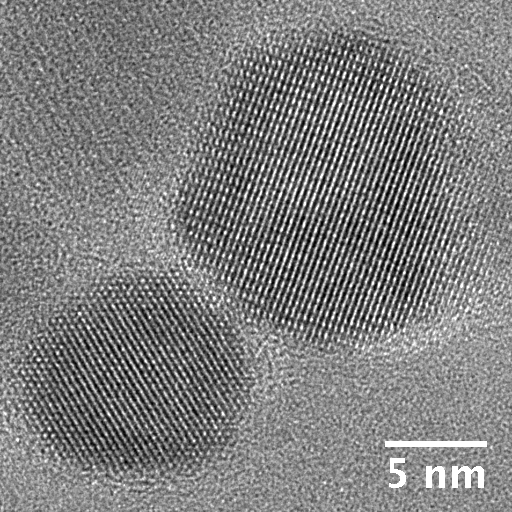

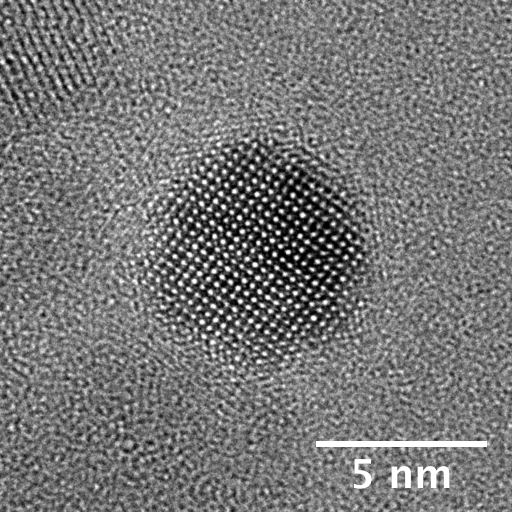

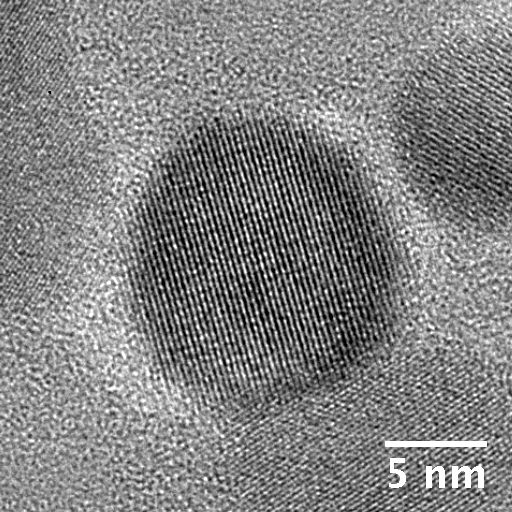

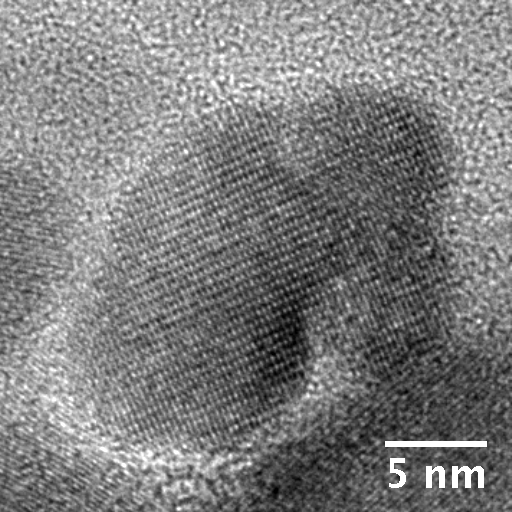

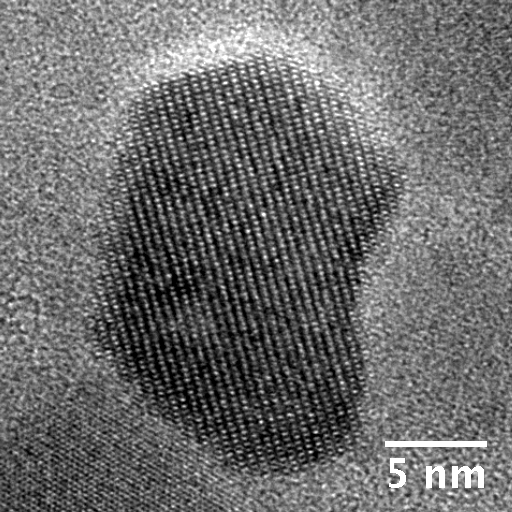

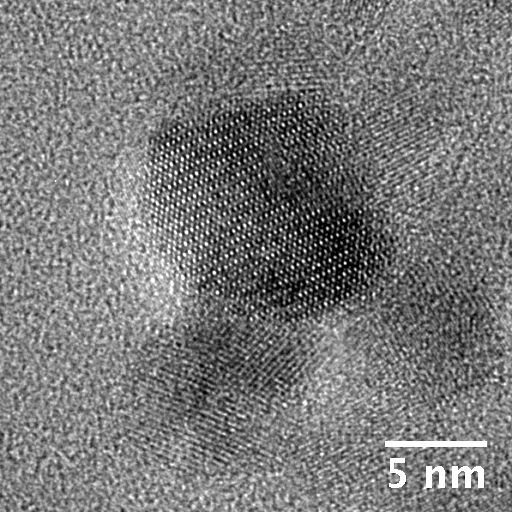

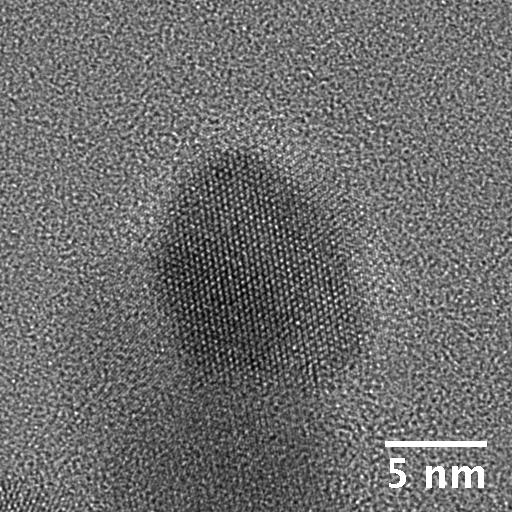

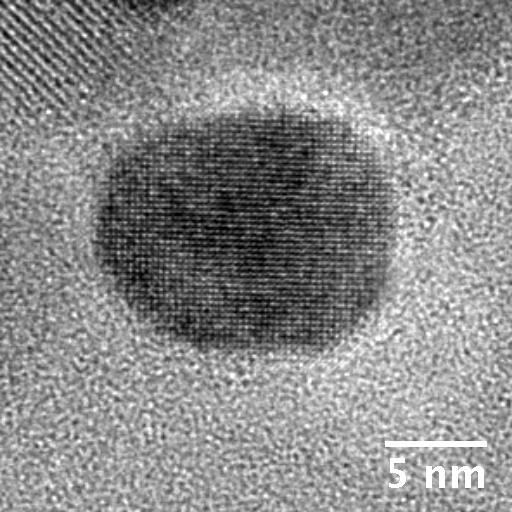

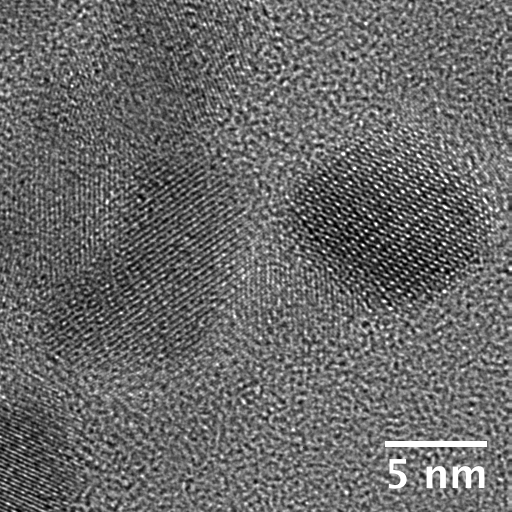


t = 0

28 days - HS

28 days - RHS

**Figure S5.** HR-TEM observations of magnetite at t=0 and after 28 days showing the conservation of the particles crystallinity.

**
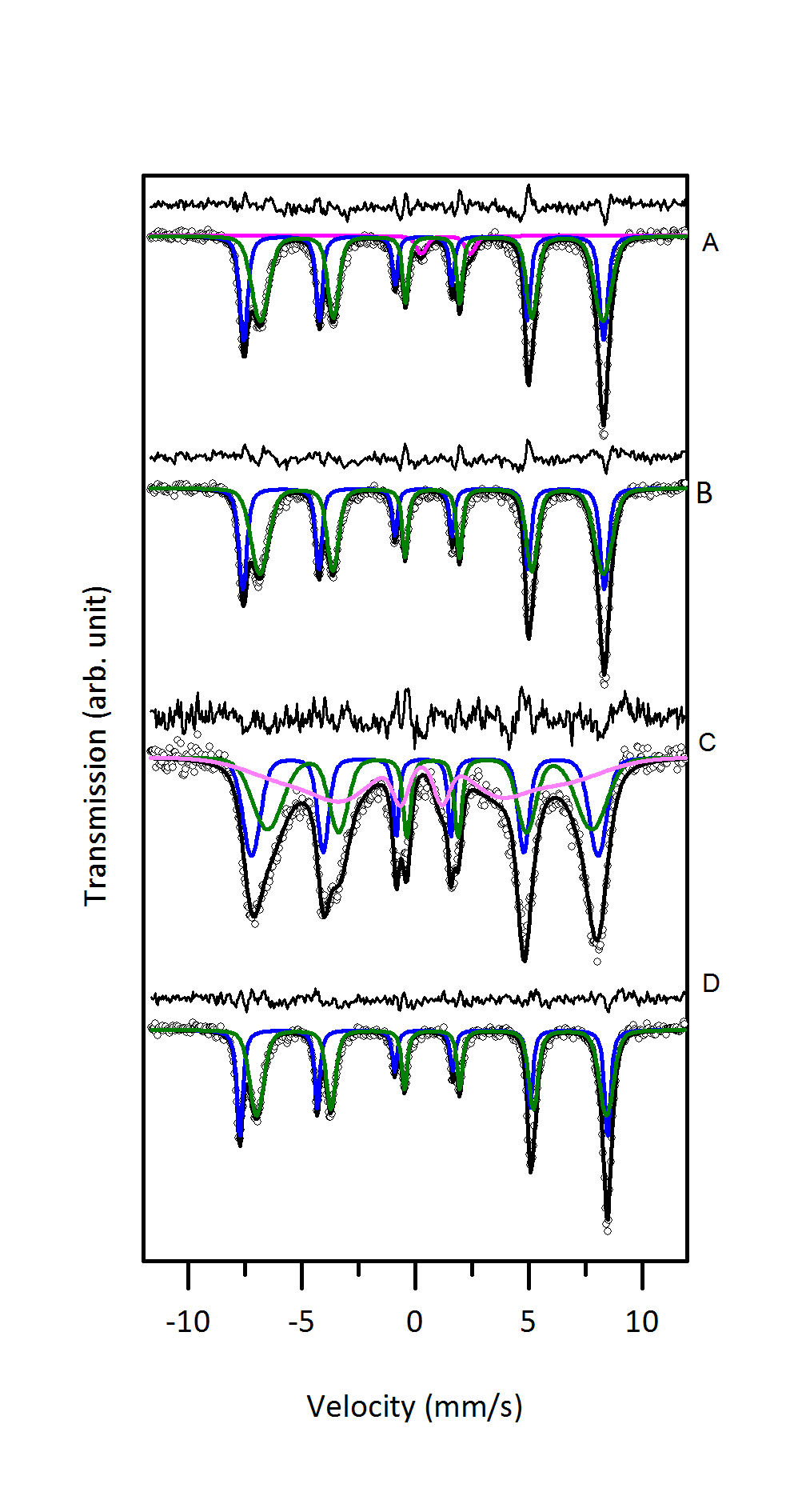
**

**Figure S6.** Mössbauer spectra of A) biogenic magnetite, B) 13 nm magnetite, C) 7 nm magnetite and D) 500 nm magnetite collected at 140 K. The open circles correspond to experimental data, black solid lines to fitted data and the blue and green lines correspond to tetrahedral A and octahedral B sites in magnetite. The magenta line correspond to siderite and the light magenta to a poorly crystalline fraction. The stacked black lines correspond to the residuals.

**Table S1.** Mössbauer spectroscopy fittings of the starting material for the 4 types of magnetite with spectra collected at 140K.

|  |  | **CS** | **ΔE_Q_** | **H** | **Population** | **χ^2^** |
| --- | --- | --- | --- | --- | --- | --- |
| **Sample** |  | **(mm/s)** | **(mm/s)** | **(T)** | **(%)** |  |
| Biomag. | Siderite | 1.34 | 2.15 |  | 4.0**±**0.5 | 1.94871 |
|  | Site A | 0.36 | 0.01 | 49.2 | 38.6**±**0.7 |  |
|  | Site B | 0.74 | -0.04 | 46.9 | 57.4**±**0.8 |  |
|  |  |  |  |  |  |  |
| 13 nm mag. | Site A | 0.36 | 0.01 | 49.4 | 38.4**±**0.7 | 2.02101 |
|  | Site B | 0.74 | -0.03 | 47.0 | 61.6**±**0.7 |  |
|  |  |  |  |  |  |  |
| 7 nm mag. | Site A | 0.39 | 0.06 | 47.4 | 29.3**±**2.3 | 1.08914 |
|  | Site B | 0.70 | -0.13 | 44.4 | 35.4**±**2.6 |  |
|  | Paramagnetic fraction | 0.38 | 0.24 | 35.4 | 35.2**±**3.3 |  |
|  |  |  |  |  |  |  |
| 500 nm mag. | Site A | 0.37 | 0.00 | 50.3 | 39.1**±**0.9 | 1.00255 |
|  | Site B | 0.73 | -0.03 | 47.8 | 60.9**±**0.9 |  |

The following abbreviations are used: center shift (CS), Quadropole splitting (ΔE_Q_), average hyperfine field (H) and relative intensity of each component (Population).

**Table S2.** Particle size analysis as determined by µXRD and TEM micrographs.

| **Sample** | **Diameter (nm)^a^** | **Diameter (nm)^b^** |
| --- | --- | --- |
| Biomag., t=0 | 11.4 | 13.6±2.1 |
| Biomag., t=2 months | 11.2 | - |
| Biomag., + HS, t=2 months | 10.8 | 12.4±2.2 |
| Biomag., + RHS t=7 days | - | 12.8±1.9 |
| Biomag., + RHS t=2 months | 10.9 | 12.8±2.0 |
|  |  |  |
| 13 nm mag., t=0 | 12.1 | 13.2±2.4 |
| 13 nm mag., t=2 months | 12.2 | - |
| 13 nm mag. + HS, t=7 days | - | 12.9±2.4 |
| 13 nm mag. + HS, t=2 months | 9.4 | 13.4±3.2 |
| 13 nm mag, + RHS, t=2 months | 8.1 | 13.2±2.4 |
|  |  |  |
| 7 nm mag,, t=0 | 6.6 | 7.1±1.2 |
| 7 nm mag,, t=2 months | 5.2 | - |
| 7 nm mag, + HS, t=2 months | 4.8 | 6.7±1.2 |
| 7 nm mag, + RHS, t=2 months | 5.6 | 7.2±1.1 |
|  |  |  |
| 500 nm mag., t=0 | - | 524±156 |

^a^Determined via µXRD.

^b^Determined from particle size analysis of TEM micrographs.

- Not determined.

**Table S3.** Fe(II) and Fe(III) concentrations in the dissolved and solid-phase for the initial and end magnetite samples with native and reduced HS.

|  | **Initial concentrations (mM)** | | | | **End concentrations (mM)** | | | |
| --- | --- | --- | --- | --- | --- | --- | --- | --- |
| **Sample** | **Fe^2+^ (aq)** | **Fe^3+^ (aq)** | **Fe(II) (s)** | **Fe(III) (s)** | **Fe^2+^ (aq)** | **Fe^3+^ (aq)** | **Fe(II) (s)** | **Fe(III) (s)** |
| Biomag. + HS | 1.696 ± 0.00390 | 0.118 ± 0.0404 | 2.071 ± 0.146 | 5.767 ± 0.560 | 2.792 ± 0.300 | 3.469 ± 1.089 | 1.070 ± 0.264 | 2.570 ± 0.686 |
| Biomag. +RHS | 1.737 ± 0.147 | 0.0673 ± 0.00490 | 1.979 ± 0.0626 | 5.349 ± 0.0715 | 4.036 ± 0.155 | 2.592 ± 0.0985 | 0.885 ± 0.0987 | 2.102 ± 0.236 |
| 13 nm mag. + HS | 0.973 ± 0.0418 | 0.110 ± 0.0136 | 1.821 ± 0.191 | 4.968 ± 0.576 | 1.100 ± 0.341 | 0.677 ± 0.144 | 2.364 ± 0.0541 | 7.127 ± 0.209 |
| 13 nm mag. +RHS | 1.028 ± 0.0207 | 0.0809 ± 0.0110 | 1.994 ± 0.236 | 5.215 ± 0.566 | 1.078 ± 0.112 | 0.734 ± 0.157 | 2.481 ± 0.163 | 6.361 ± 0.279 |
| 7 nm mag. + HS | 0.497 ± 0.0128 | 1.072 ± 0.0890 | 1.361 ± 0.0410 | 6.016 ± 0.441 | 1.474 ± 0.281 | 4.173 ± 1.057 | 0.976 ± 0.113 | 5.180 ± 0.592 |
| 7 nm mag. +RHS | 0.646 ± 0.0890 | 1.184 ± 0.341 | 1.354 ± 0.673 | 7.143 ± 0.576 | 1.558 ± 0.320 | 4.045 ± 0.403 | 0.987 ± 0.0769 | 4.714 ± 0.371 |
| 500 nm mag. + HS | 0.0697 ± 0.0134 | 0.00437 ± 0.000899 | 3.582 ± 0.253 | 7.546 ± 1.014 | 0.0855 ± 0.0102 | 0.00330 ± 0.00107 | 3.863 ± 0.110 | 8.459 ± 0.291 |
| 500 nm mag. +RHS | 0.135 ± 0.0196 | 0.00191 ± 0.00160 | 3.680 ± 0.096 | 7.891 ± 0.285 | 0.138 ± 0.0230 | 0.00417 ± 0.00247 | 3.781 ± 0.0478 | 8.342 ± 0.239 |

**Table S4.** Mössbauer spectroscopy fittings of the 4 types of magnetite in the absence of HS at t=0 and t=2 months and after 2 months incubation with HS or RHS.

| **Sample** | **A** | **B** | **Fe(II)/Fe(III)** |
| --- | --- | --- | --- |
| Biomag., t=0 | 38.6**±**0.7 | 57.4**±**0.8 | 0.43**±**0.01 |
| Biomag., t= 2 months | 39.2**±**0.6 | 50.0**±**0.7 | 0.39**±**0.01 |
| Biomag., + HS, t=2 months | 40.4**±**1.2 | 57.2**±**1.3 | 0.41**±**0.02 |
| Biomag., + RHS t=2 months | 33.4**±**2.3 | 61.3**±**2.4 | 0.48**±**0.04 |
| 13 nm mag., t=0 | 38.4**±**0.7 | 61.6**±**0.7 | 0.44**±**0.01 |
| 13 nm mag., t=2 months | 40.1**±**0.8 | 59.9**±**0.8 | 0.43**±**0.01 |
| 13 nm mag. + HS, t=2 months | 43.8**±**0.8 | 56.2**±**0.8 | 0.39**±**0.01 |
| 13 nm mag. + RHS, t=2 months | 39.6**±**1.6 | 60.4**±**1.6 | 0.43**±**0.02 |
| 7 nm mag., t=0 | 49.0**±**3.5 | 33.8**±**2.9 | 0.26**±**0.03 |
| 7 nm mag., t=2 months | 45.2**±**2.5 | 27.1**±**2.0 | 0.23**±**0.02 |
| 7 nm mag. + HS, t=2 months | 44.2**±**2.1 | 29.0**±**2.2 | 0.25**±**0.02 |
| 7 nm mag. + RHS, t=2 months | 45.2**±**2.5 | 27.1**±**2.0 | 0.23**±**0.02 |
| 500 nm mag., t=0 | 39.1**±**0.9 | 60.9**±**0.9 | 0.44**±**0.01 |
| 500 nm mag., t=2 months | 41.0**±**1.0 | 59.0**±**1.0 | 0.42**±**0.01 |
| 500 nm mag. + HS, t=2 months | 39.5**±**0.6 | 60.5**±**0.6 | 0.43**±**0.01 |
| 500 nm mag. + RHS, t=2 months | 38.6**±**0.7 | 61.4**±**0.7 | 0.44**±**0.01 |

**Table S5.** Concentrations (% or µM) of solid Fe(II), aqueous Fe^2+^, total Fe and total Fe(II) used for electron transfer calculations.

|  | **Fe(II) (%)** | | **Fe^2+^ (%)** | | **Fe_tot_ (µM)** | | **Fe(II)_tot._ (µM)** | |
| --- | --- | --- | --- | --- | --- | --- | --- | --- |
| **Sample** | **0h** | **28 days** | **0h** | **28 days** | **0h** | **28 days** | **0h** | **28 days** |
| Biomag. + HS | 21.5 | 10.9 | 17.6 | 28.1 | 9646.1 | 9901.2 | 3766.9 | 3862.0 |
| Biomag. + RHS | 21.8 | 9.2 | 19.1 | 42.0 | 9092.2 | 9614.5 | 3715.6 | 4921.0 |
| 13 nm mag. + HS | 23.1 | 20.5 | 12.4 | 9.4 | 7871.9 | 11549.9 | 2794.2 | 3464.2 |
| 13 nm mag. + RHS | 24.0 | 23.3 | 12.4 | 10.1 | 8305.7 | 10655.2 | 3021.5 | 3559.7 |
| 7 nm mag. + HS | 15.2 | 8.3 | 5.6 | 12.4 | 8946.2 | 11802.9 | 1858.7 | 2449.5 |
| 7 nm mag. + RHS | 13.1 | 8.7 | 6.3 | 13.8 | 10328.4 | 11304.1 | 2000.9 | 2544.2 |
| 500 nm mag. + HS | 32.1 | 31.1 | 0.6 | 0.7 | 11199.1 | 12409.4 | 3652.1 | 3947.8 |
| 500 nm mag. + RHS | 31.4 | 30.8 | 1.2 | 1.1 | 11703.4 | 12259.2 | 3815.0 | 3918.2 |
|  |  |  |  |  |  |  |  |  |
| Biomag. |  | 26.7 |  | 9.1 |  | 9103.1 |  | 3253.0 |
| 13 nm mag. |  | 25.0 |  | 2.8 |  | 11934.6 |  | 3320.1 |
| 7 nm mag. |  | 15.0 |  | 1.5 |  | 11213.7 |  | 1855.2 |
| 500 nm mag. |  | 32.5 |  | 0.2 |  | 10278.3 |  | 3368.2 |
